# Structural Characterization and Anti-Inflammatory Activity of Polysaccharides from *Tremella fuciformis* on Monosodium Urate-Stimulated RAW264.7 Macrophages

**DOI:** 10.3390/foods12244398

**Published:** 2023-12-07

**Authors:** Wei Deng, Li Wu, Zheng Xiao, Yibin Li, Zhipeng Zheng, Shouhui Chen

**Affiliations:** 1 College of Food Science, Fujian Agriculture and Forestry University, Fuzhou 350002, China; ld199905@163.com (W.D.); zzp9701@163.com (Z.Z.); 2 Institute of Food Science and Technology, Fujian Academy of Agricultural Sciences, Fuzhou 350003, China; xxj1963@163.com (L.W.); ethyxwat@163.com (Z.X.); cshh1307@163.com (S.C.); 3 National Research and Development Center of Edible Fungus Processing Technology, Fuzhou 350003, China; 4 Key Laboratory of Subtropical Characteristic Fruits, Vegetables and Edible Fungi Processing (Coconstruction by Ministry and Province), Ministry of Agriculture and Rural Affairs, Fuzhou 350003, China; 5 Fujian Key Laboratory of Agricultural Product (Food) Processing, Fuzhou 350003, China

**Keywords:** *Tremella fuciformis*, polysaccharides, transcriptomics, hypoxia inducible factor-1 signaling pathway, anti-inflammatory activity

## Abstract

The structural characteristics and anti-inflammatory activity of *Tremella fuciformis* polysaccharides (TFPs) were investigated. The study showed that TFPs were mainly composed of mannose, rhamnose, glucuronic acid, glucose, galactose, xylose, and fucose. TFPs significantly inhibited monosodium urate (MSU)-induced inflammation of RAW264.7 cells, as well as the secretion levels of TNF-α, IL-1β, and IL-18 cytokines. The concentrations of malondialdehyde and reactive oxygen species in RAW264.7 macrophages were reduced, but superoxide dismutase activity was increased. RNA-Seq technology was applied to explore the mechanisms of TFPs ameliorating MSU-induced inflammation of RAW264.7 macrophages. Results revealed that TFPs significantly reduce MSU-stimulated inflammatory damage in RAW 264.7 cells by inhibiting signaling pathways like the hypoxia inducible factor-1 (HIF-1) signaling pathway and erythroblastic oncogene B (ErbB) signaling pathway. This study provides a foundation for TFPs to be developed as novel anti-inflammatory drugs.

## 1. Introduction

Epidemiologic evidence suggests that the incidence of gout is rising dramatically worldwide in the 21st century [1]. Gout is a recurrent inflammatory disease caused by an abnormal purine biosynthesis metabolism, resulting in elevated levels of uric acid in the blood. This excess uric acid leads to the deposition of urate crystals in various parts of the human body, including the joints, kidneys, and cardiovascular system, causing conditions such as arthritis, renal disease, and cardiovascular disease [2]. The main component of urate is monosodium urate (MSU), which plays a crucial role in the inflammatory process. MSU activates chemotaxis of inflammatory cells, accelerates the inflammatory cascade, and promotes the synthesis and release of inflammatory cytokines (such as interleukin (IL) IL-6, IL-β, and tumor necrosis factor (TNF)-α) and mediators (such as NO). These factors contribute to the progression of inflammation and further worsen the symptoms of gout [3,4]. Therefore, the treatment of inflammation in gout is a critical and challenging task, and cell experiments in vitro can provide a theoretical basis for the treatment of gout in vivo.

Mushroom polysaccharides, macromolecular polymers commonly found in nature, have the advantages of being safe and highly effective, and are widely employed as anti-tumor [5], anti-diabetes [6], and anti-inflammatory agents [7]. Favorable anti-inflammatory activity has been reported for mushroom polysaccharides by stimulating the immune system (macrophage function) and modulating cytokine expression. The potential anti-inflammatory mechanism of this substance may involve the inhibition of the MAPK and PI 3K/Akt signaling pathways, resulting in a decrease in the production of NO, IL-6, IL-β and TNF-α [8].

*Tremella fuciformis* is a nutritious edible mushroom that is widely cultivated in the provinces of Sichuan, Yunnan, and Fujian in China. It is a popular food and herbal ingredient in China owing to its abundance of protein, polysaccharides, dietary fiber, and other essential nutrients, including vitamins, trace elements, and minerals [9]. In ancient China, *T. fuciformis* was highly valued for its medicinal properties, believed to moisturize the lungs, relieve coughs, nourish the body, boost the constitution, and improve overall health [10]. *T. fuciformis* polysaccharides (TFPs) are considered to be the primary active components related to health. Bioactivity studies have focused on various physiological functions, including their antioxidant, antitumor, and antiaging properties [11], Notably, there is potential for TFPs’ immunomodulatory activity to display anti-inflammatory qualities. Further research is required to better understand TFPs’ anti-inflammatory effects. The aim of this investigation was to isolate and characterize acidic polysaccharides from *T. fuciformis* and to evaluate their anti-inflammatory properties on RAW 264.7 cells stimulated by MSU. The anti-inflammatory cytokine levels and oxidative stress markers malondialdehyde (MDA), superoxide dismutase (SOD), and reactive oxygen species (ROS) were measured to determine the anti-inflammatory efficacy of TFPs. Furthermore, a transcriptomics analysis was conducted to investigate the likely molecular mechanisms of TFPs’ anti-inflammatory properties. Our research will provide new perspectives on the influence of TFPs on anti-inflammatory effects.

## 2. Materials and Methods

### 2.1. Materials

*T. fuciformis* Tr01 was obtained from Xiangyun Biotechnology Development Co., Ltd., (Fuzhou, China). RAW264.7 cells were obtained from the cell bank of the Chinese Academy of Sciences (Shanghai, China). All chemicals and reagents used were of analytical grade.

### 2.2. Preparation of TFPs

The extraction of TFPs was performed according to Song but with some modifications [12]. *T. fuciformis* powder (25 g) was mixed with 500 mL of water at 95 °C for 2 h. The extract was obtained by filtration using 150 mesh gauze. The obtained concentration of water-soluble extract was concentrated to 10% of its original volume at 70 °C using a rotary evaporator, and the obtained concentrated solution was thoroughly mixed with five times the volume of anhydrous ethanol for 24 h. The sediment obtained was then filtered using 100 mesh gauze. The alcohol in the sediment was removed by hot air drying. The sediment was deproteinized using the Sevage method, and TFPs were acquired after lyophilization.

### 2.3. Preparation of MSU

Briefly, 10 mg/mL MSU was prepared according to the reference with some modifications [13]. An amount of MSU was weighed and dried at 180 °C until constant weight, after which the dried MSU was weighed and added to DMEM medium to prepare an MSU solution with a mass concentration of 10 mg/mL.

### 2.4. Fourier Transformed Infrared (FT-IR) Spectroscopy

Two milligrams of TFPs was mixed and ground with 100 mg of anhydrous KBr in a mortar and pestle, and then compressed into a transparent disk using a compressor. The FT-IR spectrum was obtained with an FT-IR (iCAN9, Tianjin Nenpu Science Co., Ltd., Tianjin, China) over the range of 4000–400 cm^−1^.

### 2.5. Molecular Weight Monosaccharide Composition Determination and Nuclear Magnetic Resonance of TFPs

The average molecular weight (Mw) and number average molecular weight (Mn) of TFPs were determined by a high-performance gel permeation chromatography (HPGPC) system (Waters Co., Ltd., Milford, MA, USA) as previously reported [14]. Polydispersity coefficients (PDI = Mw/Mn) were calculated.

High-performance liquid chromatography (HPLC) was used for the determination of the monosaccharide compositions of TFPs as previously reported but with some modifications [15]. A 100 μL volume of TFPs (5 g/L) was thoroughly mixed with 100 μL of trifluoroacetic acid solution (4 mol/L) and dissolved at 121 °C for 2 h. After cooling, 200 μL of methanol was added to remove trifluoroacetic acid, and the sample was finally blown dry with nitrogen gas. The role of methanol was to eliminate trifluoroacetic acid via an esterification reaction, leading to the formation of ester compounds that can be dried more easily by blowing nitrogen gas. Then, the sample was dissolved in aseptic water to be analyzed by HPLC. The equipment consisted of an Agilent 1100 system (Agilent Technologies, Santa Clara, CA, USA) which was equipped with ZORBAX Eclipse XDB-C18 (250 mm × 4.6 mm, 5 μm). The column temperature was set to 30 °C, the mobile phase was 0.1 M phosphate (pH 6.7) buffer-acetonitrile (83:17, V/V), injection volume 20 μL, detection wavelength 250 nm, and flow rate 1 mL/min. The HPLC chromatograms of standard monosaccharides are shown in Figure S1.

For nuclear magnetic resonance, 50 mg of TFPs was dissolved in 0.6 mL of deuterium oxide (D_2_O). The ^1^H-NMR and ^13^C-NMR data were collected using an NMR spectrometer (AVIII-850M, Bruker, Fällanden, Switzerland).

### 2.6. Cell Culture

RAW264.7 cells were cultured in Dulbecco’s modified eagle medium (DMEM) (containing 10% fetal bovine serum, 100 U/mL penicillin, 100 μg/mL streptomycin) at 37 °C and 5% CO_2_. The RAW264.7 cells were divided into 6 groups (Table S1): the control group (CK), the MSU group (M), the TFPs-treated groups (TFPs-20, TFPs-40, TFPs-60) and the positive control group (PC).

### 2.7. Cell Viability Analysis

The medium was replaced with 100 μL of DMEM containing 10 μL of Cell Counting Kit-8 (CCK-8) solution and incubated in the dark at 37 °C for 2 h. After removal of the supernatant, the absorbance of each well was measured at 450 nm using an enzyme marker.

### 2.8. Determination of Cytokine Levels by ELISA

RAW264.7 cells were cultivated in 24-well plates, then the supernatant was removed after 6 h of incubation. Three replicate wells of each group were incubated, and the total volume of each well was 1 mL. The intracellular cytokine production (TNF-α, IL-1β, and IL-18) was measured with ELISA kits according to the manufacturer’s instructions.

### 2.9. ROS Determination by Fluorescence Spectrophotometer

The ROS produced by the cells were determined according to the a slightly modified reference method [16]. The intracellular ROS production was measured by dichlorodihydrofluorescein diacetate (DCFH-DA) assay according to the manufacturer’s instructions. A 100 µM final concentration of DCFH-DA was incorporated for 30 min, after incubation, the cells underwent centrifugation to remove the excess dye and medium. They were then resuspended in 100 μL of PBS before analysis by a fluorescence microscope with a maximum excitation wavelength of 488 nm and a maximum emission wavelength of 525 nm.

### 2.10. Determination of SOD and MDA

The contents of MDA and SOD were determined by published procedures [17]. After removal of the supernatant, the intracellular MDA and SOD were measured with kits according to the manufacturer’s instructions.

### 2.11. Determination of Cytokine mRNA Expression

Total RNA was extracted according to instructions. The primer sequences of cytokines are listed in the Table S2. The obtained RNA was amplified using QuantStudio™ 6 Flex Real-Time Fluorescent Quantitative PCR System (Applied Biosystems, Waltham, MA, USA). Then, the mRNA expression levels of cytokines were evaluated following the literature [18].

### 2.12. RNA-Seq Analysis

The transcriptomic analysis of RAW264.7 cells was conducted based on the published method [19]. Total RNA was extracted and enriched using magnetic beads with Oligo (dT) for cDNA library construction at Beijing AllweGene Technology Co., Ltd. (Beijing, China). Sequencing was performed on an Illumina PE150 platform (San Diego, CA, USA). Differential gene expression under different conditions was evaluated using DE-Seq R software, (1.10.1) and log_2_ (fold change) and *p*-values were calculated for each differential gene. The samples of differentially expressed genes (DEGs) were also subjected to KEGG and GO enrichment analysis using GOseq software (version 1.10.1). The KEGG pathway was analyzed for the significant enrichment of DEGs by applying the hypergeometric test.

### 2.13. Statistical Analysis

Data are presented as mean ± standard deviation (*n* = 5). Statistical analysis was performed with SPSS software (version 21.0, IBM, Chicago, IL, USA), and *p* < 0.05 was considered statistically significant. Graphs were drawn using GraphPad Prism software (version 9.5.0, GraphPad software, San Diego, CA, USA).

## 3. Results

### 3.1. Characterization of TFPs

**Figure 1:**
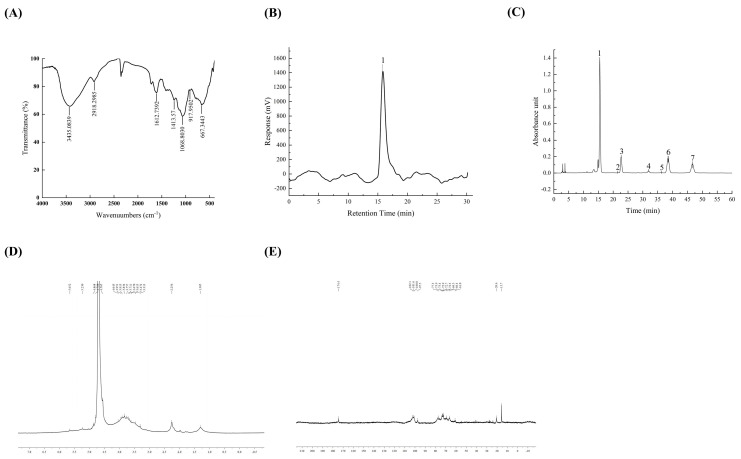
FT-IR spectra of TFPs (**A**). GPC-RI profiles of TFPs (**B**). The HPLC chromatograms of component monosaccharides released from TFP (**C**) (peaks 1~7 were mannose, rhamnose, glucuronic acid, glucose, galactose, xylose, and fucose, respectively). NMR spectra of TFPs, ^1^H NMR (**D**), and ^13^C NMR (**E**).

Figure 1A illustrates the FT-IR spectrum of TFPs showing bands at 3435 and 2918 cm^−1^, corresponding to the stretching vibrations of O-H and C-H groups, respectively. A weak peak at 1612 cm^−1^ confirmed the presence of hydrated hydroxyl groups, which was ascribed to asymmetric stretching modes of the COO group. A peak at 1068 cm^−1^ suggested the stretching vibrations of the pyranose ring in the structure of TFPs, and the characteristic peak at 917 cm^−1^ indicated the existence of β-configuration glycosidic bonds. These results were in agreement with the previous study [20], establishing the existence of functional polysaccharide groups in TFPs.

The purity and molecular weight of TFPs were examined using HPGPC. The results suggested that TFPs had a large molecular and uniform composition. The Mw measurement obtained from the standard regression equation was found to be 9.245 × 10^5^ Da (Figure 1B and Table 1), which is lower than the value reported in recent studies [21]. Mw might be posited as a crucial factor having an impact on the physicochemical and functional properties of polysaccharides, such as stability, solution viscosity and biological activity [22]. The PDI displayed associations with the Mw distribution characteristics of the polysaccharides. The PDI of the TFPs was 1.215, indicating significant uniformity. The results were consistent with a previous study [23].

Table 2 and Figure 1C illustrate the monosaccharide composition of TFPs. The results indicated that the monosaccharides of TFPs consisted of mannose (56.88%), glucuronic acid (11.66%), xylose (16.23%), fucose (12.69%), glucose (1.98%), galactose (0.32%), and rhamnose (0.23%). According to previous reports, the monosaccharides found in TFPs are generally composed of mannose, xylose, fucose and glucuronic acid [24,25]. It is worth noting that the content of gluconic acid was positively related to the antioxidant activity [24]. In this study, the content of glucuronic acid in TFPs increased by 33% in comparison with that reported in a previous report [25], indicating their potential to enhance anti-inflammatory activity related to antioxidant biological activity [26].

The structures of TFPs were further analyzed by 1D ^1^H NMR and ^13^C NMR. The TFPs displayed narrow regions between 3.3~5.5 ppm in the ^1^H NMR (Figure 1D) spectrum, which was indicative of polysaccharides. Among them, four anomeric proton signals were observed at 5.23 ppm, 4.79 ppm, 4.57 ppm and 4.87 ppm, indicating that the sugar residues of TFPs contained β and α configurations. In the ^13^C NMR spectrum (Figure 1E), the main anomeric carbon signals were located at 102.1 ppm, 101.6 ppm, 100.8 ppm, and 97.5 ppm. They were assigned to α-D-Glcp and β-D-Manp residues. A weak signal at 174.4 ppm indicated that the TFPs may contain glucuronic acid. From the structural analysis, combined with the monosaccharide composition, the main chain of TFPs may consist mainly of (1,4)-α-D-Glcp and (1,6)-β-D-Manp.

### 3.2. Effect of TFPs on Cell Viability

**Figure 2:**
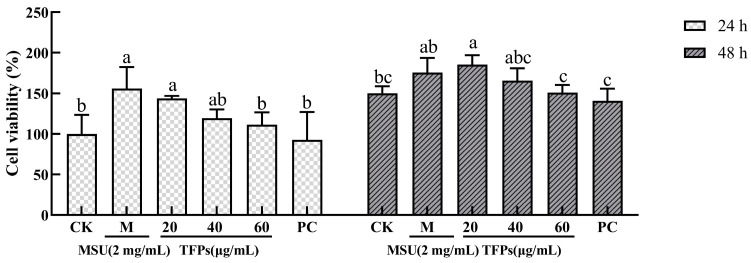
The viability of RAW264.7 cells (*n* = 5). Data are expressed as mean ± standard error. Different letters indicate significant differences (*p* < 0.05) between each treatment.

The cell viability of the TFPs-20 and TFPs-40 groups showed no obvious change (compared to the MSU group) at 24 h and 48 h (*p* > 0.05), but that of TFPs-60 group showed obvious change at 24 h and 48 h (*p* < 0.05) (Figure 2). The results showed that MSU could activate the proliferation of RAW264.7 cells in a concentration-dependent manner. The reason may be that MSU could bind to the toll-like receptor (TLR) on the cell surface and activate phagocytosis, while 60 μg/mL of TFPs could inhibit this process [27].

### 3.3. Effect of TFPs on Cytokine Secretion

**Figure 3:**
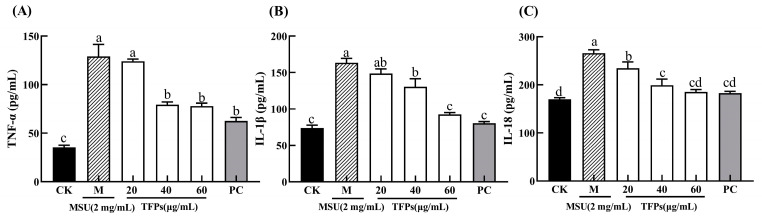
The levels of TNF-α (**A**), IL-1β (**B**) and IL-18 (**C**) secreted by RAW264.7 cells. Different letters indicate significant differences (*p* < 0.05) between each treatment.

The levels of cytokine in the CK group were significantly lower than those in the M group (*p* < 0.05). With the addition of TFPs, cytokine levels in the TFPs group were significantly reduced, and there was a significant difference between TFPs-20 and TFPs-60, with 60 μg/mL of TFPs showing the most significant reduction (*p* < 0.05) (Figure 3). The results suggested that MSU could stimulate the secretion of cytokines, and TFPs significantly inhibited the secretion of cytokines in a concentration dependent manner.

### 3.4. Effect of TFPs on Oxidative Stress Levels

**Figure 4:**
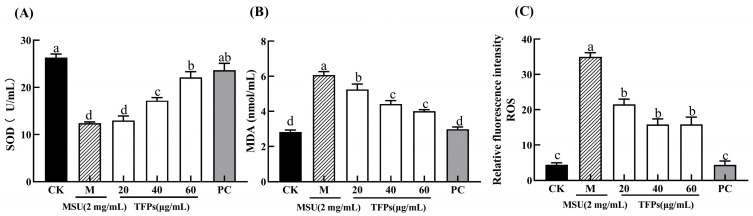
The levels of SOD (**A**), MDA (**B**) and ROS (**C**) in RAW264.7 cells (*n* = 5). Different letters indicate significant differences (*p* < 0.05) between each treatment.

**Figure 5:**
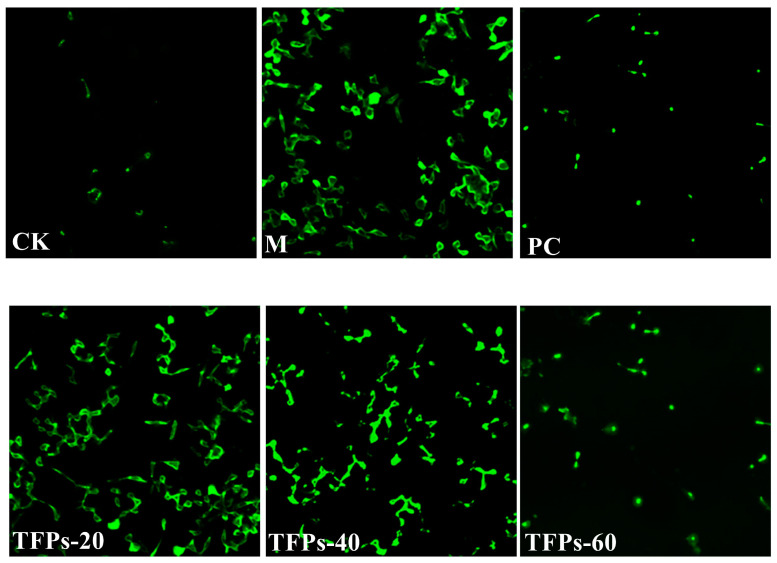
ROS changes in RAW264.7 cells. The darker the color, the more ROS the cell produces.

In general, ROS is a natural byproduct of oxygen metabolism, and MDA is the final breakdown product of membrane lipid peroxidation, reflecting the cellular antioxidant capacity and the degree of cellular damage. In general, they both are at low levels in the cells. The relative fluorescence intensity of ROS and the levels of MDA in the M group were significantly higher than those in the CK group (*p* < 0.05) (Figure 4). This indicated that MSU can significantly increase the oxidative damage of cells. The relative fluorescence intensity of ROS and the level of MDA in TFP groups were significantly decreased compared to the M group. Additionally, the activity of SOD enzyme was significantly higher in the TFP groups than that in the M group, and there was a significant difference between TFPs-20 and TFPs-60 (*p* < 0.05). The results indicated that TFPs could significantly inhibit the oxidative stress in RAW264.7 cells. ROS alterations were observed under flow cytometry (Figure 5); the figure also clearly demonstrates the differences in ROS between the groups.

### 3.5. Effect of TFPs on mRNA Expression

**Figure 6:**
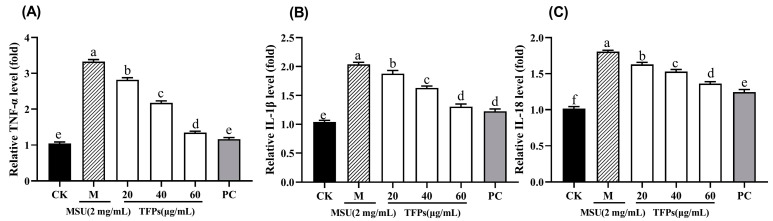
The mRNA expression of TNF-α (**A**), IL-1β (**B**) and IL-18 (**C**) in RAW264.7 cells (*n* = 5). Different letters indicate significant differences (*p* < 0.05) between each treatment.

The mRNA levels of TNF-α, IL-1β, and IL-18 in cells of the M group were significantly higher than those in the CK group (*p* < 0.05). The addition of TFPs caused significant concentration-dependent reductions in mRNA expression levels (*p* < 0.05) (Figure 6). The findings indicated that TFPs could effectively inhibit the mRNA expression of cytokines. The findings suggested that TFPs had anti-inflammatory properties, inhibiting the release of cytokines by down-regulating the expression of their corresponding mRNA and thereby mitigating the inflammation.

### 3.6. Analysis of Differentially Expressed mRNAs

Transcriptome analysis using RNA-Seq was performed to further understand the mechanism of the anti-inflammatory activity of TFPs. Nine samples were analyzed for sequencing data quality, yielding 205,018,120 raw reads. The error rate of sequencing was found to be only 0.03% after the raw reads were filtered as clean reads. This indicated that the sequencing results met the requirements and could be analyzed further.

**Figure 7:**
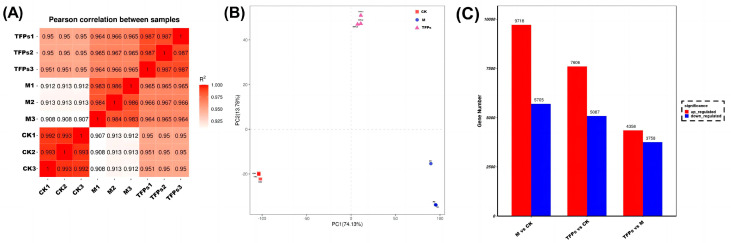
The changes in the sample correlation (**A**), principal component analysis; different colors represent different groups in the figure (**B**), and differential gene statistics among the M group, CK group and the TFPs-60 group (**C**).

The heat map of RNA-Seq correlation displayed a high similarity between samples, with correlation coefficients ranging from 0.907 to 0.993, thus indicating the experiment’s reliability (Figure 7A). Principal component analysis (PCA) was performed to evaluate the differences among groups and sample replicates within each group. Smaller differences between the same groups indicated that the experiments were well parallelized and the results were more reliable. The disparity between the TFPs and M groups was more pronounced, implying that TFPs treatment elicited alterations in gene expression of the macrophages (Figure 7B).

The data statistics for DEGs are shown in Figure 7C. Overall, A higher number of DEGs indicated that MSU and TFPs interventions were very effective. Compared with the CK group, there were a total of 12,693 DEGs in the TFPs-60 group (7606 up-regulated genes and 5087 down-regulated genes), and 15,423 DEGs in the M group (9718 up-regulated genes and 5705 down-regulated genes). In contrast, there were 8116 DEGs in the TFPs group compared to the M group (4358 up-regulated genes and 3758 down-regulated genes).

### 3.7. GO Enrichment Analysis

**Figure 8:**
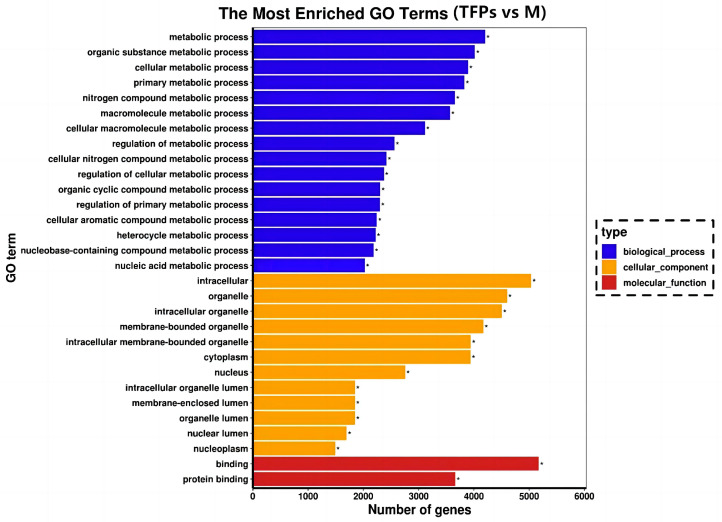
Gene Ontology enrichment analysis of the differential genes. With “*” indicating the significantly enriched GO terms.

Gene Ontology (GO) is frequently employed for functionally categorizing genes in the field of bioinformatics, which covers cellular components (CC), molecular functions (MF), and biological processes (BP) [28]. Figure 8 displays the 30 most important GO terms. Most DEGs were present in BP and CC as well, but mostly in BP. The most significant terms were “metabolic process” and “organic substance metabolic process” in the BP category, while the terms in the CC category were “intracellular” and “organelles”. In addition, the most significant terms in the MF category were “binding” and “protein binding”. Many of these GO terms were associated with inflammatory responses and interleukin production in RAW264.7 cells.

### 3.8. KEGG Pathway Enrichment Analysis

**Figure 9:**
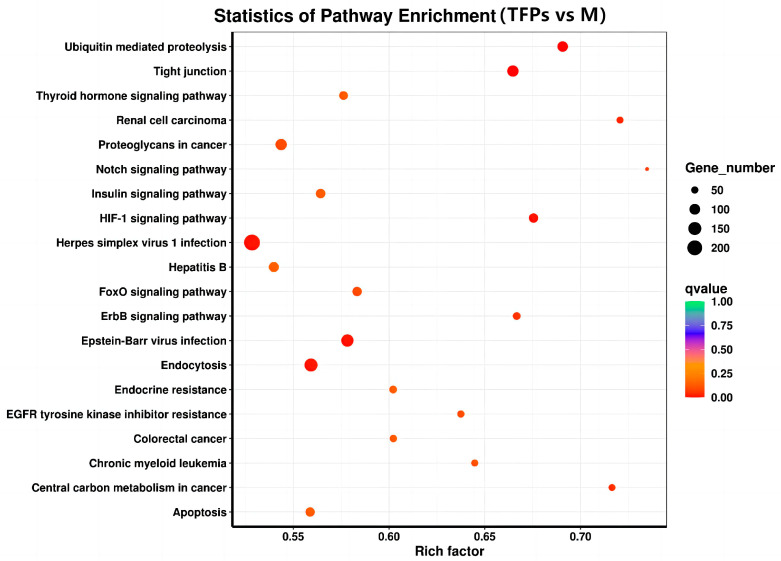
The Kyoto Encyclopedia of Genes and Genomes pathway enrichment analysis of differential genes.

Kyoto Encyclopedia of Genes and Genomes (KEGG) is a database that integrates genomic, chemical, and systemic functional information to reflect systematic, genomic, chemical, and health information [29]. To identify potential signaling pathways closely related to the anti-inflammatory activity of TFPs, KEGG enrichment analysis was investigated. The KEGG enrichment analysis was graphically represented by scatter plots (Figure 9). The hypoxia inducible factor-1(HIF-1) signaling pathway and erythroblastic oncogene B (ErbB) signaling pathway were more significantly enriched among the top 20 pathways. There were 77 DEGs (43 up-regulated DEGs and 34 down-regulated ones.) and 64 DEGs (36 up-regulated DEGs and 20 down-regulated ones.) enriched in the HIF-1 and ErbB signaling pathways, respectively. The findings indicated that TFPs may exert their anti-inflammatory effects by inhibiting the HIF-1 and ErbB signaling pathways.

## 4. Discussion

In general, natural mushroom polysaccharides have anti-inflammatory properties. Several studies have demonstrated that mushroom polysaccharides can induce the activation and differentiation of phagocytic cells, modulate the levels of immune factors and cytokines, and exert regulatory effects on anti-inflammatory pathways. Examples of such mushroom polysaccharides are *Tuber magnatum Pico* polysaccharide [30], *Boletus impolitus Fr* polysaccharide [31] and *Helvella leucopus* polysaccharide [32]. All of these polysaccharides contain a significant amount of glucuronic acid, which has been linked to anti-inflammatory properties [33]. Our study found that TFPs can alleviate the inflammation of RAW 264.7 cells induced by MSU, and their anti-inflammatory effect might be related to their characteristic structure, which includes monosaccharide compositions and glycosidic linkages [34]. Polysaccharides containing high levels of fucose and glucuronic acid tend to have significant anti-inflammatory properties [35]. The fucose and glucuronic acid contents in TFPs are as high as 12.69% and 11.66%, respectively, which may give TFPs better anti-inflammatory activity. Glycosidic linkages of the polysaccharides are another important factor that affects the polysaccharides’ anti-inflammatory activities. Previous studies demonstrated that B-(1 → 6) glycosidic linkages play significant roles in enhancing anti-inflammatory effects [36]. (1,6)-β-D-Manp is in the main chain of TFPs, which may give TFPs better anti-inflammatory activity. TFPs significantly reduced cytokine concentrations and their relative mRNA cytokine levels with a dose–effect relationship to some extent. This finding is consistent with the conclusions published in [37]. Additionally, TFPs effectively reduced TNF-α and IL-6 production in RAW 264.7 cells.

In the last 10 years, RNA-Seq has become an important tool to explore signaling pathways. RNA-Seq was used to perform a global screen of all transcripts in RAW 264.7 cells stimulated with M and TFPs-60. A total of 15,423 DEGs between the M and TFPs-60 groups were obtained, including 9718 up-regulated and 5705 down-regulated genes. The analysis of the results indicated that the experiments had good parallelism. GO functional annotations of DEGs between M and TFPs-60 groups showed that the highest number of DEGs were enriched biological processes in the GO terms, including “metabolic process”, “cell metabolic process”, and “external stimulus”. Among them, “protein binding” indicated that small molecule proteins may play a role in various biological activities. KEGG enrichment analysis was employed to identify the signaling pathways involved in the effect of TFPs on macrophages. The ErbB signaling pathway and HIF-1 signaling pathway exhibited a negative correlation with anti-inflammatory activity among the top 20 enriched pathways (*p*-value < 0.05). In recent years, research on inflammation-related signaling pathways has primarily focused on nuclear factor-κB (NF-κB) and mitogen-activated protein kinase (MAPK). Numerous studies have been demonstrated that polysaccharides possess anti-inflammatory properties in macrophages by stimulating the secretion of inflammatory factors through the activation of MAPK and NF-κB pathways, as well as by interacting with toll-like receptors [38]. However, the number of studies on the anti-inflammatory mechanisms of other signaling pathways is limited. Whether there is a connection between other signaling pathways, or whether multiple signaling pathways are activated at the same time, still needs to be further investigated.

HIF-1 consists of HIF-1α and HIF-1β subunits; HIF-1α is stabilized in the cytoplasm under hypoxic conditions. It combines with HIF-1β subunit to form a complete HIF-1, which enters into the nucleus to function as nuclear transcription factor [39]. There are over 100 known downstream regulatory genes or factors of HIF-1, which are distributed in almost all cells. They include vascular endothelial growth factor (VEGF), cyclooxygenase 2 (COX-2), erythropoietin (EPO), etc. Among others, they regulate various cellular functions, including cell proliferation and apoptosis, immunomodulation, and inflammation [40]. HIF-1α has been reported to be an important drug target for controlling and attenuating the development of the inflammatory response [41]. An excess of ROS can upregulate HIF-1 α expression, which in turn promotes inflammation and increases the production of inflammatory factors. This creates a feedback loop that stimulates HIF-1 expression [42]. It is widely believed that hypoxia aggravates the inflammatory response primarily through the HIF-1 signaling pathway. The inflammatory reaction further aggravates the hypoxia due to the release of numerous exudates. Therefore, blocking activation of the HIF-1 pathway may serve to reduce oxidative stress levels and the ability of molecules to activate responses and inhibit subsequent inflammatory processes. TFPs could inhibit the activity of the HIF-1 signaling pathway, thereby reducing the expression of inflammatory factors and effectively alleviating inflammation.

The ErbB signaling pathway is a complex network that regulates various cellular processes, including growth, differentiation, survival and migration. One of its components, the ErbB-1 receptor, has the ability to modulate the transcription and expression of genes involved in inflammation via the MAPK pathway [43]. The MAPK pathway includes three subgroups (ERK, p38, and JNK) [44], which are called “upstream milestones” in inflammation and apoptosis. Activation of MAPK signaling pathways could increase the secretion of inflammatory factors and is directly involved in the control of proinflammatory cytokines, which are important factors in inflammation. Inhibiting MAPK can effectively improve inflammation. TFPs could down-regulate the expression of the ErbB receptor family, thereby interfering with the MAPK pathway and reducing the levels of proinflammatory mediators. The attenuation of the MAPK pathway and the reduction of protein phosphorylation in this pathway could lower the levels of proinflammatory mediators. Consequently, TFPs may inhibit the activity of the ErbB signaling pathway, thereby alleviating inflammation.

Notably, AKT2 showed downregulation in the signaling pathways. Recently, studies have found that AKT2 can modulate inflammatory responses; the inhibition of inflammation in mice was observed upon pharmacological blocking of AKT2 [45]. Furthermore, miRNA-200b treatment in a mouse model of ulcerative colitis-related colorectal cancer was shown to attenuate inflammatory responses through the blockade of the AKT2-mediated NF-κB/IL-6 signaling pathway, which is considered to be the molecular mechanism of the inhibition of inflammation [46]. The finding illustrated that TFPs inhibited inflammatory responses through the expression of TNF-α, IL-18, and IL-1β in MSU-induced RAW264.7 cells. There was a significant reduction in the levels of inflammation factors in the cells (relative to the MSU group). These results indicated that TFPs could effectively hinder downstream inflammatory responses by inhibiting the activation of HIF-1 and ErbB signaling pathways. Consequently, further investigations into the anti-inflammatory effects of TFPs both in vivo and in vitro are crucial for the scientific treatment of gout-induced inflammation.

## 5. Conclusions

In this study, TFPs were extracted and purified from *T. fuciformis* for anti-inflammatory activity evaluation. TFPs mainly consist of a macromolecular polysaccharide with an average molecular weight of 9.245 × 10^5^ Da, which is composed of seven monosaccharides including mannose, rhamnose, glucuronic acid, etc. TFPs comprise characteristic structures and functional groups of polysaccharides such as pyran rings and hydroxyl groups. TFPs could effectively inhibit the secretion of inflammatory factors and reduce the levels of oxidative stress in the gout model RAW 264.7 cell induced with MSU. KEGG analysis indicated that the potential mechanism of TFPs alleviated inflammation in RAW264.7 cells and might inhibit activation of the HIF-1 and ErbB signaling pathways. Overall, this work innovatively investigated the anti-inflammatory biological activity and potential molecular mechanisms of TFPs, which would lay the foundation for their further development as drugs for treating gout in animal research.

## Figures and Tables

**Table 1:** Molecular weight distribution of TFPs.

	Retention Time (min)	Mw (Da)	Mn (Da)	Mw/Mn	Ratio (%)
TFPs	14.97~18.20	9.245 × 10^5^	7.609 × 10^5^	1.215	100

**Table 2:** Monosaccharide composition of TFPs.

Monosaccharide Composition	Molar Ratio (%)
Mannose	56.88
Aminoglucose	ND
Ribose	ND
Rhamnose	0.23
Glucuronic acid	11.66
Galacturonic acid	ND
Aminogalactose	ND
Glucose	1.98
Galactose	0.32
Xylose	16.23
Arabinose	ND
Fucose	12.69

ND: Not detected or lower than limit of quantification.

## Data Availability

Data is contained within the article or Supplementary Materials.

## Supplementary Materials

The following supporting information can be downloaded at: https://www.mdpi.com/article/10.3390/foods12244398/s1, Table S1: Details of drug treatment for each group; Table S2: Primer sequences used for qRT-PCR. Figure S1: The HPLC chromatograms of standard monosaccharides.

## References

[1] Piao W., BO Y.C., Zhao L.Y., Yu D.M. (2022). Status of serum uric acid and hyperuricemia among adults in China: China nutrition and health surveillance (2015). Biomed. Environ. Sci..

[2] Danve A., Sehra S.T., Neogi T. (2021). Role of diet in hyperuricemia and gout. Best Pract. Res. Clin. Rheumatol..

[3] Gu H., Yu H., Qin L., Yu H., Song Y., Chen G., Zhao D., Wang S., Xue W., Wang L. (2023). MSU crystal deposition contributes to inflammation and immune responses in gout remission. Cell Rep..

[4] Wang Y., Zhu W., Lu D., Zhang C., Wang Y. (2021). Tetrahydropalmatine attenuates MSU crystal-induced gouty arthritis by inhibiting ROS-mediated NLRP3 inflammasome activation. Int. Immunopharmacol..

[5] Li Z., Wang M., Yang Z. (2023). Structural characterization, anti-tumor and immunomodulatory activity of intracellular polysaccharide from *Armillaria luteo-virens*. Carbohydr. Res..

[6] Khursheed R., Singh S.K., Wadhwa S., Gulati M., Awasthi A. (2020). Therapeutic potential of mushrooms in diabetes mellitus: Role of polysaccharides. Int. J. Biol. Macromol..

[7] Muszyńska B., Grzywacz-Kisielewska A., Kała K., Gdula-Argasińska J. (2018). Anti-inflammatory properties of edible mushrooms: A review. Food Chem..

[8] Li W., Cai Z., Mehmood S., Wang Y., Pan W., Zhang W., Lu Y., Chen Y. (2018). Polysaccharide FMP-1 from *Morchella esculenta* attenuates cellular oxidative damage in human alveolar epithelial A549 cells through PI3K/AKT/Nrf2/HO-1 pathway. Int. J. Biol. Macromol..

[9] Wen L., Gao Q., Ma C., Ge Y., You L., Liu R.H., Fu X., Liu D. (2016). Effect of polysaccharides from *Tremella fuciformis* on UV-induced photoaging. J. Funct. Foods.

[10] Ge X., Huang W., Xu X., Lei P., Sun D., Xu H., Li S. (2020). Production, structure, and bioactivity of polysaccharide isolated from *Tremella fuciformis* XY. Int. J. Biol. Macromol..

[11] Zhao X., Hu Y., Wang D., Guo L., Yang S., Fan Y., Zhao B., Wang Y., Abula S. (2011). Optimization of sulfated modification conditions of tremella polysaccharide and effects of modifiers on cellular infectivity of NDV. Int. J. Biol. Macromol..

[12] Song Q., Jiang L., Yang X., Huang L., Yu Y., Yu Q., Chen Y., Xie J. (2019). Physicochemical and functional properties of a water-soluble polysaccharide extracted from Mung bean (*Vigna radiate* L.) and its antioxidant activity. Int. J. Biol. Macromol..

[13] Liu Y., Zhu H., Zhou W., Ye Q. (2020). Anti-inflammatory and anti-gouty-arthritic effect of free Ginsenoside Rb1 and nano Ginsenoside Rb1 against MSU induced gouty arthritis in experimental animals. Chem. Biol. Interact..

[14] Bai C.L., Chen R.Z., Tan L., Bai H.L., Tian L., Lu J., Gao M., Sun H., Chi Y. (2022). Effects of multi-frequency ultrasonic on the physicochemical properties and bioactivities of polysaccharides from different parts of ginseng. Int. J. Biol. Macromol..

[15] Xu J., Zou Y., Guo L., Lin J., Jiang Z., Zheng Q. (2023). Rheological and microstructural properties of polysaccharide obtained from the gelatinous *Tremella fuciformis* fungus. Int. J. Biol. Macromol..

[16] Cianciosi D., Forbes-Hernandez T.Y., Alvarez-Suarez J.M., Ansary J., Quinzi D., Amici A., Navarro-Hortal M.D., Esteban-Munoz A., Quiles J.L., Battino M. (2021). Anti-inflammatory activities of Italian Chestnut and Eucalyptus honeys on murine RAW 264.7 macrophages. J. Funct. Foods.

[17] Naseri E., Ahmadi A. (2022). A review on wound dressings: Antimicrobial agents, biomaterials, fabrication techniques, and stimuli-responsive drug release. Eur. Polym. J..

[18] Lee J., Lee J.H., Min B., Kim K., Ahn D.U., Paik H. (2022). Immunostimulatory effect of egg yolk phosvitin phosphopeptides produced by high-temperature and mild-pressure pretreatment and enzyme combinations in RAW 264.7 cells via TLR2/MAPK signaling pathway. J. Funct. Foods.

[19] Wei H., Wang Y., Li W., Qiu Y., Hua C., Zhang Y., Guo Z., Xie Z. (2022). Immunomodulatory activity and active mechanisms of a low molecular polysaccharide isolated from Lanzhou lily bulbs in RAW264.7 macrophages. J. Funct. Foods.

[20] Xu X., Chen A., Ge X., Li S., Zhang T., Xu H. (2020). Chain conformation and physicochemical properties of polysaccharide (glucuronoxylomannan) from Fruit Bodies of *Tremella fuciformis*. Carbohyd. Polym..

[21] Lan X., Wang Y., Deng S., Zhao J., Wang L., Yao K., Jia D. (2021). Physicochemical and rheological properties of *Tremella fuciformis* polysaccharide fractions by ethanol precipitation. CyTA-J. Food.

[22] Wang W., Shen M., Jiang L., Song Q., Liu S., Xie M., Xie J. (2019). Rheological behavior, microstructure characterization and formation mechanism of *Mesona blumes* polysaccharide gels induced by calcium ions. Food Hydrocoll..

[23] Li Y., Chen J., Lai P., Tang B., Wu L. (2020). Influence of drying methods on the physicochemical properties and nutritional composition of instant *Tremella fuciformis*. Food Sci. Technol..

[24] Li P., Jiang Z., Sun T., Wang C., Chen Y., Yang Z., Du B., Liu C. (2018). Comparison of structural, antioxidant and immuno-stimulating activities of polysaccharides from *Tremella fuciformis* in two different regions of China. Int. J. Food Sci. Technol..

[25] Lin C., Tsai S. (2019). Differences in the moisture capacity and thermal stability of *Tremella fuciformis* polysaccharides obtained by various drying processes. Molecules.

[26] Islam M., Prottay A.A.S., Sultana I., Al Faruq A., Bappi M.H., Akbor M.S., Asha A.I., Hossen M.M., Machado P.E.M., Secundo Junior I.J. (2023). Phytochemical screening and evaluation of antioxidant, anti-inflammatory, antimicrobial, and membrane-stabilizing activities of different fractional extracts of *Grewia nervosa* (Lour.) Panigrahi. Food Biosci..

[27] Chen B., Li H., Ou G., Ren L., Yang X., Zeng M. (2019). Curcumin attenuates MSU crystal-induced inflammation by inhibiting the degradation of IκBα and blocking mitochondrial damage. Arthritis Res. Ther..

[28] Huang D.W., Sherman B.T., Lempicki R.A. (2009). Bioinformatics enrichment tools: Paths toward the comprehensive functional analysis of large gene lists. Nucleic Acids Res..

[29] Feng G., Huang S., Liu Y., Xiao F., Liu J., Zhang Z., Chen Q., Mao Y., Cao X., Wang Y. (2018). The transcriptome analyses of *Tagetes erecta* provides novel insights into secondary metabolite biosynthesis during flower development. Gene.

[30] Beara I.N., Lesjak M.M., Četojević-Simin D.D., Marjanović Ž.S., Ristić J.D., Mrkonjić Z.O., Mimica-Dukić N.M. (2014). Phenolic profile, antioxidant, anti-inflammatory and cytotoxic activities of black (*Tuber aestivum* Vittad.) and white (*Tuber magnatum* Pico) truffles. Food Chem..

[31] Wan H., Liu D., Yu X., Sun H., Li Y. (2015). A Caco-2 cell-based quantitative antioxidant activity assay for antioxidants. Food Chem..

[32] Zhang W., Gong L., Zhou Z., Sun M., Li Y., Sun J., Chen Y. (2022). Structural characterization and immunomodulatory activity of a mannan from *Helvella leucopus*. Int. J. Biol. Macromol..

[33] Chen H.X., Zhang M., Xie B.J. (2004). Quantification of uronic acids in tea polysaccharide conjugates and their antioxidant properties. J. Agric. Food Chem..

[34] Li N., Wang C., Georgiev M.I., Bajpai V.K., Tundis R., Simal-Gandara J., Lu X., Xiao J., Tang X., Qiao X. (2021). Advances in dietary polysaccharides as anticancer agents: Structure-activity relationship. Trends Food Sci. Technol..

[35] Liu C., Dai K., Ji H., Jia X., Liu A. (2022). Structural characterization of a low molecular weight *Bletilla striata* polysaccharide and antitumor activity on H22 tumor-bearing mice. Int. J. Biol. Macromol..

[36] Wang Z.J., Luo D.H., Liang Z.Y. (2004). Structure of polysaccharides from the fruiting body of *Hericium erinaceus* Pers. Carbohydr. Polym..

[37] Wu Y., Wei Z., Zhang F., Linhardt R.J., Sun P., Zhang A. (2019). Structure, bioactivities and applications of the polysaccharides from *Tremella fuciformis* mushroom: A review. Int. J. Biol. Macromol..

[38] Sheng K., Wang C., Chen B., Kang M., Wang M., Liu K., Wang M. (2021). Recent advances in polysaccharides from *Lentinus edodes* (Berk.): Isolation, structures and bioactivities. Food Chem..

[39] Wang Y., Li Z., Teng M., Liu J. (2021). Dihydroartemisinin inhibits activation of the AIM2 inflammasome pathway and NF-kappa B/HIF-1 alpha/VEGF pathway by inducing autophagy in A431 human cutaneous squamous cell carcinoma cells. Int. J. Med. Sci..

[40] Song X., Xing W., Zhang X., Wang X., Ji J., Lu J., Yu B., Ruan M. (2023). Exploring the synergic mechanism of *Ligusticum striatum* DC. and borneol in attenuating BMECs injury and maintaining tight junctions against cerebral ischaemia based on the HIF-1α/VEGF signalling pathway. J. Ethnopharmacol..

[41] Jiang T., Ji C., Cheng X., Gu S., Wang R., Li Y., Zuo J., Han J. (2021). Alpha-mangostin alleviated HIF-1 alpha-mediated angiogenesis in rats with adjuvant-induced arthritis by suppressing aerobic glycolysis. Front. Pharmacol..

[42] Liu Y., Xiang D., Zhang H., Yao H., Wang Y., Barreto E. (2020). Hypoxia-inducible factor-1: A potential target to treat acute lung injury. Oxidative Med. Cell. Longev..

[43] Miyagi H., Hiroshima M., Sako Y. (2021). Cell-to-cell diversification in ERBB-RAS-MAPK signal transduction that produces cell-type specific growth factor responses. Biosystems.

[44] Li J., Chen J., Huang P., Cai Z., Zhang N., Wang Y., Li Y. (2023). The anti-inflammatory mechanism of flaxseed linusorbs on lipopolysaccharide-induced RAW 264.7 macrophages by modulating TLR4/NF-κB/MAPK pathway. Foods.

[45] Reyes-Gordillo K., Shah R., Arellanes-Robledo J., Cheng Y., Ibrahim J., Tuma P.L. (2019). Akt1 and Akt2 Isoforms Play Distinct Roles in Regulating the Development of Inflammation and Fibrosis Associated with Alcoholic Liver Disease. Cells.

[46] Deng S., Wang H., Fan H., Zhang L., Hu J., Tang Q., Shou Z., Liu X., Zuo D., Yang J. (2018). Over-expressed miRNA-200b ameliorates ulcerative colitis-related colorectal cancer in mice through orchestrating epithelial-mesenchymal transition and inflammatory responses by channel of AKT2. Int. Immunopharmacol..

