# Supplementary material for: Structural Characterization and Anti-Inflammatory Activity of Polysaccharides from Tremella fuciformis on Monosodium Urate-Stimulated RAW264.7 Macrophages
Source: Foods. 2023 Dec 7;12(24):4398. doi: 10.3390/foods12244398 (PMC10743196; doi:10.3390/foods12244398)
Supplement: Supplementary file 1 [file foods-12-04398-s001.zip › foods-2648272-supplementary.pdf]

Table S1 Details of drug treatment for each group

Group	MSU	TFPs	Colchicine
CK	-	-	-
M	2 mg/mL	-	-
TFPs-20	2 mg/mL	20 μg/mL	-
TFPs-40	2 mg/mL	40 μg/mL	-
TFPs-60	2 mg/mL	60 μg/mL	-
PC	2 mg/mL	-	0.4 μg/mL

Note: The control group (CK), the MSU group (M), the TFPs-treated groups (TFPs-20, TFPs-40, TFPs-60) and the positive control group (PC).

Table S2 Primer sequences used for *qRT-PCR*

Primer	Primer sequences (5'-3')
TNF-α	F: AGGCACTCCCCCAAAAGATG
	R: CCACTTGGTGGTTTGTGAGTG
IL-1β	F: TGGGTACTGGAGAGTGGTCA
	R: GGCTTGGGAGTGAAGAGGTC
IL-18	F: CCAGTGGCTGCAGATTCAGA
	R: CTCTGCTTCGGTCCCAACAT

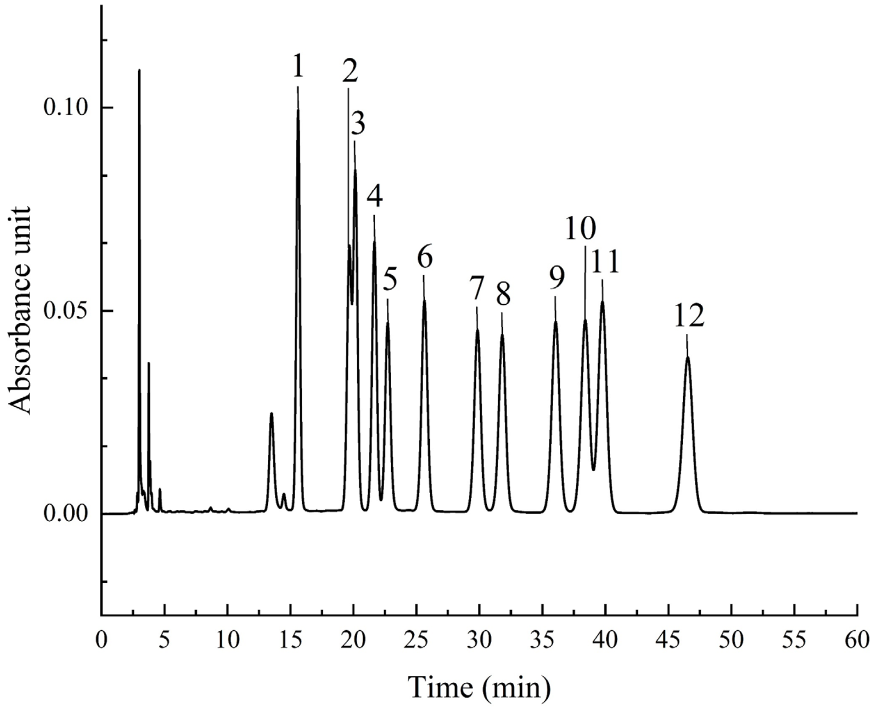

Figure S1 The HPLC chromatograms of standard monosaccharides

Note: Peak 1 ~ 12 were mannose, amino glucose, ribose, rhamnose, glucuronic acid, galacturonic acid, amino galactose, glucose, galactose, xylose, arabinose, fucose.
